# Evaluation of adhesion forces of *Staphylococcus aureus* along the length of *Candida albicans* hyphae

**DOI:** 10.1186/1471-2180-12-281

**Published:** 2012-11-27

**Authors:** Ekaterina S Ovchinnikova, Bastiaan P Krom, Henk J Busscher, Henny C van der Mei

**Affiliations:** 1 Department of Biomedical Engineering, University of Groningen and University Medical Center Groningen, Antonius Deusinglaan 1, Groningen, AV, 9713, The Netherlands; 2 Present address: Department of Preventive Dentistry (room 12 N51), Academic Centre for Dentistry Amsterdam (ACTA), University of Amsterdam and Free University Amsterdam, Gustav Mahlerlaan 3004, Amsterdam, LA, 1081, The Netherlands

**Keywords:** Bacteria, Yeast, Interaction, Adhesion forces, AFM

## Abstract

**Background:**

*Candida albicans* is a human fungal pathogen, able to cause both superficial and serious, systemic diseases and is able to switch from yeast cells to long, tube-like hyphae, depending on the prevailing environmental conditions. Both morphological forms of *C. albicans* are found in infected tissue, often in combination with *Staphylococcus aureus*. Although bacterial adhesion to the different morphologies of *C. albicans* has been amply studied, possible differences in staphylococcal adhesion forces along the length of *C. albicans* hyphae have never been determined. In this study, we aim to verify the hypothesis that the forces mediating *S. aureus* NCTC8325-4^GFP^ adhesion to hyphae vary along the length of *C. albicans* SC5314 and MB1 hyphae, as compared with adhesion to yeast cells.

**Results:**

*C. albicans* hyphae were virtually divided into a “tip” (the growing and therefore youngest part of the hyphae), a “middle” and a so-called “head” region (the yeast cell from which germination started). Adhesion forces between *S. aureus* NCTC8325-4^GFP^ and the different regions of *C. albicans* SC5314 hyphae were measured using atomic force microscopy. Strong adhesion forces were found at the tip and middle regions of *C. albicans* hyphae (−4.1 nN and −4.0 nN, respectively), while much smaller adhesion forces were measured at the head region (−0.3 nN). Adhesion forces exerted by the head region were comparable with the forces arising from budding yeast cells (−0.5 nN). A similar regional dependence of the staphylococcal adhesion forces was found for the clinical isolate involved in this study, *C. albicans* MB1.

**Conclusions:**

This is the first time that differences in adhesion forces between *S. aureus* and different regions of *C. albicans* hyphae have been demonstrated on a quantitative basis, supporting the view that the head region is different from the remainder of the hyphae. Notably it can be concluded that the properties of the hyphal head region are similar to those of budding yeast cells. These novel findings provide new insights in the intricate interkingdom interaction between *C. albicans* and *S. aureus*.

## Background

*Candida albicans* is an opportunistic human pathogen and the leading cause of a wide range of human fungal infections. *C. albicans* is a polymorphic fungus and either grows as a unicellular budding yeast cell or in a filamentous, (pseudo)hyphal form, depending on environmental conditions, such as temperature, pH or presence of chemical stimuli such as serum components or N-acetylglucosamine [1-3]. The ability to switch between different morphologies is important for *C. albicans* virulence [4,5]. It is presumed that yeast cells facilitate dissemination to target organs, whereas hyphae play a role in further tissue invasion due to the ability to adhere to and pierce host epithelial and endothelial cells, damaging them through the release of hydrolytic enzymes and initiate candidiasis [5-7]. *C. albicans* morphological plasticity also plays an important role in biofilm formation and maturation. *C. albicans* mutants unable to perform morphological switches can develop only rudimentary biofilms, that are structurally less stable than wild type biofilms [8-10].

*C. albicans* co-exists with a highly diverse bacterial flora in various sites of the human body, resulting in mixed species biofilms [11,12]. For survival and reproductive success, interacting microorganisms in polymicrobial communities are involved in antagonistic or synergistic relationships. *C. albicans* is often co-isolated with *Pseudomonas aeruginosa* during catheter-associated infections or infections of patients suffering from cystic fibrosis and burn wounds [13-16]. *P. aeruginosa* can specifically adhere to *C. albicans* hyphae but not to yeast cells, which leads to rapid lysis and death of hyphae through a currently unidentified mechanism [17,18]. *C. albicans* and *Streptococcus gordonii* on the other hand, form a synergistic partnership since these streptococci enhance *C. albicans* filamentation, whereas *C. albicans* can stimulate streptococcal biofilm formation on different kind of surfaces [19].

Klotz et al. [20] showed that in approximately 11% of polymicrobial bloodstream infections, *C. albicans* was co-isolated in conjunction with *Staphylococcus aureus*. Moreover, *C. albicans* and *S. aureus* are able to form complex polymicrobial biofilms on various mucosal surfaces, and within a biofilm *S. aureus* is mostly associated with hyphal cells and not with yeast cells [21,22]. Interestingly, co-infection of mice with *C. albicans* and *S. aureus* demonstrated a synergistic effect, resulting in increased mice mortality [23,24]. Furthermore, recent *in vitro* and *in vivo* studies demonstrated that *S. aureus* may use adhesion to *C. albicans* hyphae to become invasive. Using an *ex vivo* murine tongue model, it was shown that oral co-colonization by *C. albicans* and *S. aureus* led to penetration of tongue tissue by hyphae with adhering *S. aureus*[25].

**Figure 1:**
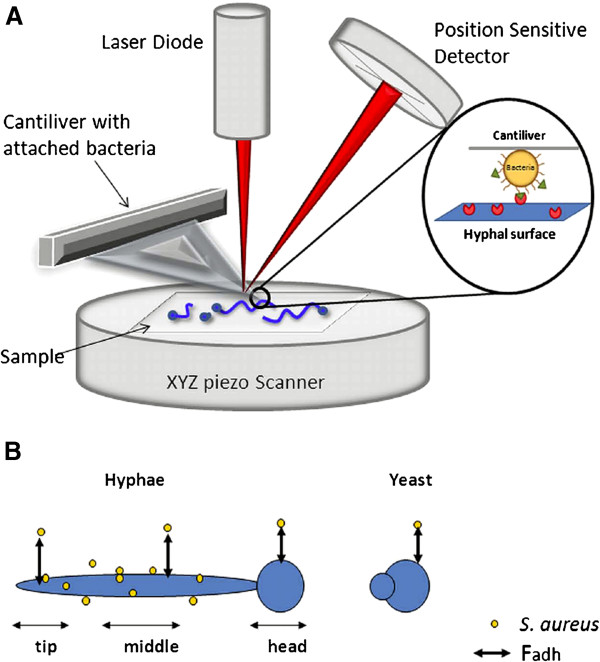
**Schematic illustration of the principle of atomic force microscopy and definition of different hyphal regions.** (**A**) Schematic presentation of AFM set-up. A sample with attached *C. albicans* cells is positioned by a xyz piezo scanner, while a bacterium attached to a tipless AFM cantilever is brought into contact with the hyphal surface. The deflection of the cantilever upon retract is a measure of the adhesion forces between a bacterium and the hyphal surface and is detected by an optical laser. The laser beam is focused on the very end of the cantilever and reflected onto a position sensitive detector from which the adhesion forces can be calculated, provided the mechanical properties of the cantilever are known. (**B**) Schematic indication of the different hyphal regions defined for bacterial-hyphal adhesion force measurements.

Atomic Force Microscopy (AFM) is a state-of-the-art technique that allows recording of the actual adhesion force that occurs between a bacterium and *C. albicans* (see Figure 1A). AFM has recently been applied to identify the nature of the adhesion forces between *P. aeruginosa* and *C. albicans*[26]. Bacterial adhesion to hyphae was always accompanied by strong adhesion forces, but did not occur to yeast cells. Poisson analyses of adhesion forces indicated that the outermost mannoprotein-layer on hyphal surfaces created favorable acid–base conditions for adhesion, allowing close approach of *P. aeruginosa.* Removal of these proteins caused unfavorable acid–base conditions, preventing adhesion of *P. aeruginosa*. Despite the notable importance of *C. albicans* morphological plasticity for bacterial-fungal interaction, possible differences in bacterial adhesion forces along the length of *C. albicans* hyphae have never been determined. Hyphae grow in a linear mode, with the tip of the hyphae representing the youngest part and the region closer to the original germinating yeast cell being the oldest. Here we hypothesize, that these differences along the length of a hypha may impact the adhesion forces with bacteria. The aim of this paper is to verify this hypothesis. To this end, we virtually divided (see Figure 1B) *C. albicans* hyphae into a “tip” (the growing end of the hyphae), a “middle” and a so-called head region (the yeast cell from which germination started) and measured actual adhesion forces that occur between these hyphal regions of two different *C. albicans* strains and a *S. aureus* strain using AFM. 

## Methods

### Strains*,* growth conditions and harvesting

*C. albicans* SC5314 (a commonly used, wild type reference strain), *C. albicans* MB1 (a biofilm-associated, clinical isolate [27]) and bacterial strain *S. aureus* NCTC8325-4 (wild type) were used*.* To generate green fluorescent protein (GFP)-expressing *S. aureus* NCTC8325-4, pMV158GFP [28] was introduced into competent bacterial cells by electroporation [29]. Selection of subsequent transformants was performed on tryptone soya broth with 1.5% bactoagar (TSB, Oxoid, Basingstoke, UK) plates containing 10 μg/mL tetracycline. *S. aureus* NCTC8325-4 that received pMV158GFP (*S. aureus* NCTC8325-4^GFP^) showed constitutive GFP expression that could be visualized using fluorescence microscopy.

Strains were grown on TSB agar plates, supplemented with tetracycline when appropriate. Single colonies were inoculated in 5 mL TSB containing 10 μg/mL tetracycline for bacterial pre-cultures or 5 mL yeast nitrogen base acids *(*YNB; Difco, Sparks, USA) pH 7, containing 0.5% D-glucose for *C. albicans* pre-cultures. *S. aureus* was routinely grown at 37°C while *C. albicans* was grown at 30°C to prevent hyphal formation for 24 h with rotation (150 rpm) and used to inoculate a main culture (1:50 dilution of pre-culture). Main bacterial cultures were grown for an additional 18 h under the same conditions. *C. albicans* hyphae were induced by growing a culture (1:50 dilution) for 4 h with rotation (150 rpm) at 37°C in 12 wells tissue culture polystyrene plates (Costar, Corning Inc., NY, USA). Hyphal formation was obtained at 90-95% efficiency under these conditions, as confirmed by phase contrast microscopy. Main cultures were harvested by centrifugation for 5 min at 6,250 x *g* and 14,800 x *g* for *S. aureus* and *C. albicans*, respectively, followed by two washes with phosphate buffered saline (PBS: 10 mM potassium phosphate, 0.15 M sodium chloride, pH 7) and resuspended in PBS.

### Adhesion of staphylococci to hyphae and yeast using fluorescence microscopy

Adhesion of *S. aureus* NCTC8325-4^GFP^ to *C. albicans* in its hyphal morphology was verified using fluorescence microscopy (Leica DM4000B, Heidelberg, Germany). After 4 h of hyphal formation, wells were washed once with PBS. Bacteria were added to a final optical density measured at 600 nm (OD_600_) of 0.1 in PBS. After 3.5 h of co-incubation with staphylococci at 37°C under static conditions, wells were gently washed two times with PBS and *C. albicans* hyphae were counter-stained with Calcofluor White (35 μg/mL, 15 min at room temperature), known to bind to chitin-rich areas of the fungal cell wall. Note that PBS was used in order to avoid the influence of growth, while co-incubation was done at 37°C in order to mimic the human body temperature. Afterwards, images were taken at five randomly chosen locations in the wells using a 40x water immersion objective using filter sets for GFP and UV. All experiments were performed in triplicate with separately grown cultures.

### Staphylococcal adhesion forces along hyphae using atomic force microscopy

Adhesion forces between *S. aureus* NCTC8325-4^GFP^ and hyphae were measured at room temperature in PBS using an optical lever microscope (Nanoscope IV, Digital Instruments, Woodbury, NY, USA) as described before [26]. Briefly, *C. albicans* was immobilized on glass slides (Menzel, GmbH, Germany), coated with positively charged poly-L-lysine. A fungal suspension was deposited onto the coated glass and left to settle at room temperature for 20 min. Non-adhering cells were removed by rinsing with demineralized water and the slide was kept hydrated prior to AFM analysis in phosphate buffer. To create a bacterial probe, *S. aureus* was immobilized onto poly-L-lysine treated tipless “V”-shaped cantilevers (DNP-0, Veeco Instruments Inc., Woodbury, NY, USA). Bacterial probes were freshly prepared for each experiment.

AFM experiments were performed at room temperature due to the limitations of the equipment. This is unlikely to have an effect on the outcome of physico-chemical measurements such as of adhesion forces, as here the absolute temperature scale, that is in Kelvin units, is relevant. On a Kelvin scale the change from 37°C to 22°C is very small, decreasing only from 293 Kelvin to 273 Kelvin.

For each bacterial probe, force curves were measured after different bond-maturation times up to 60 s on the same, randomly chosen spot on a hyphal or yeast cell with a z-scan rate of less than 1 Hz. To ensure that no bacteria detached from the cantilever during the experiment, control force-distance curves were made with 0 s contact time after each set of measurements. Whenever the “0 s contact time” forces measured deviated more than 0.5 nN from the initial measurement, a bacterial probe was considered damaged and replaced. For each combination of a bacterial strain and fungal–coated glass surface, five different probes were employed on average and the number of bacterial probes used depended on the outcome of the control measurements. Calibration of each cantilever was done using the thermal tuning method (Nanoscope V6.13r1), yielding a range of spring constants from 0.03 to 0.06 (N/m).

### Statistics

Typically, measured bacterial adhesion forces contained a large spread and were not normally distributed (Shapiro–Wilk test, P < 0.01). Hence, data are presented as median and interquartile range. Adhesion forces for different fungus-bacterium pairs were compared using non-parametric analyses (Mann–Whitney test). Differences were considered significant when the P-value was < 0.05.

## Results

### Adhesion of staphylococci to hyphae and yeast cells using fluorescence microscopy

**Figure 2:**
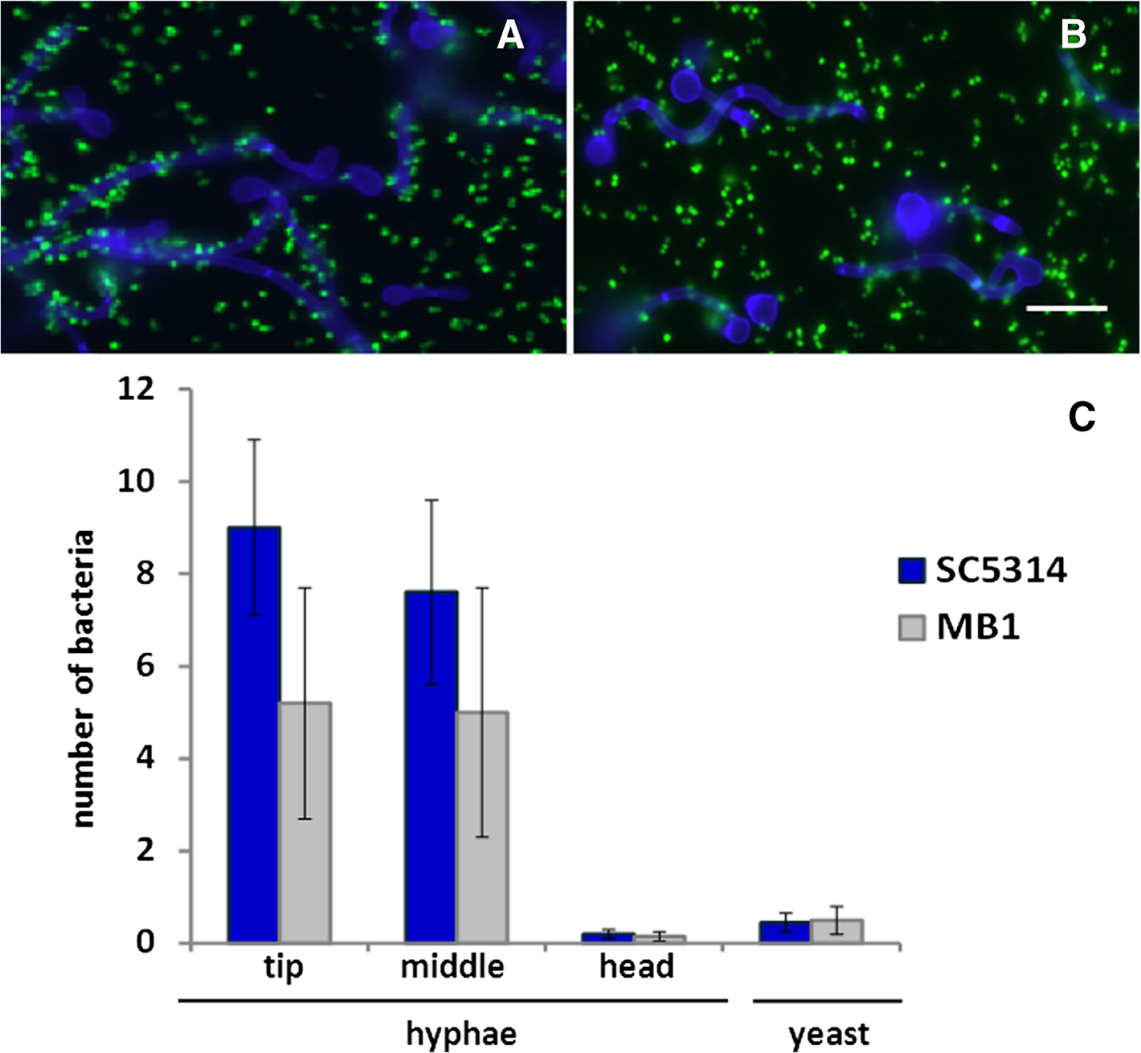
**Microscopic analysis of inter-species interaction.** Examples of fluorescent microscopic images and quantitative enumeration of the interaction between *S. aureus* NCTC8325-4^GFP^ and *C. albicans* strains. (**A**) *S. aureus* with *C. albicans* SC5314 hyphae. (**B**) *S. aureus* with *C. albicans* MB1 hyphae. Scale bar corresponds with 10 μm. (**C**) number of *S. aureus* NCTC8325-4^GFP^ adhering per 10 μm length of different regions of *C. albicans* hyphae and yeast cells. Error bars represent SD over three experiments with separately cultured organisms and involving 30 hyphae per bacterium-fungus pair.

In order to assess the adhesion of *S. aureus* NCTC8325-4^GFP^ along the length of *C. albicans* hyphae, we used two different fungal strains: *C. albicans* SC5314 and *C. albicans* MB1. Bacterial adhesion to hyphae was visualized with fluorescent microscopy and quantitated by enumeration of adhering bacteria per unit hyphal length (Figure 2). Most bacteria adhered to the tip and middle regions of the hyphae and adhered only scarcely to the head region of the hyphae or to non-germinating yeast cells (Figure 2C). Note that strictly speaking, a comparison of the number of staphylococci adhering per unit hyphal length may not be directly compared with the number of bacteria adhering to a non-germinating yeast cell. Both *C. albicans* strains showed the same trend, although bacteria adhered to *C. albicans* SC5314 in higher numbers than to the clinical isolate MB1. 

### Adhesion force along the hyphae using atomic force microscopy

**Figure 3:**
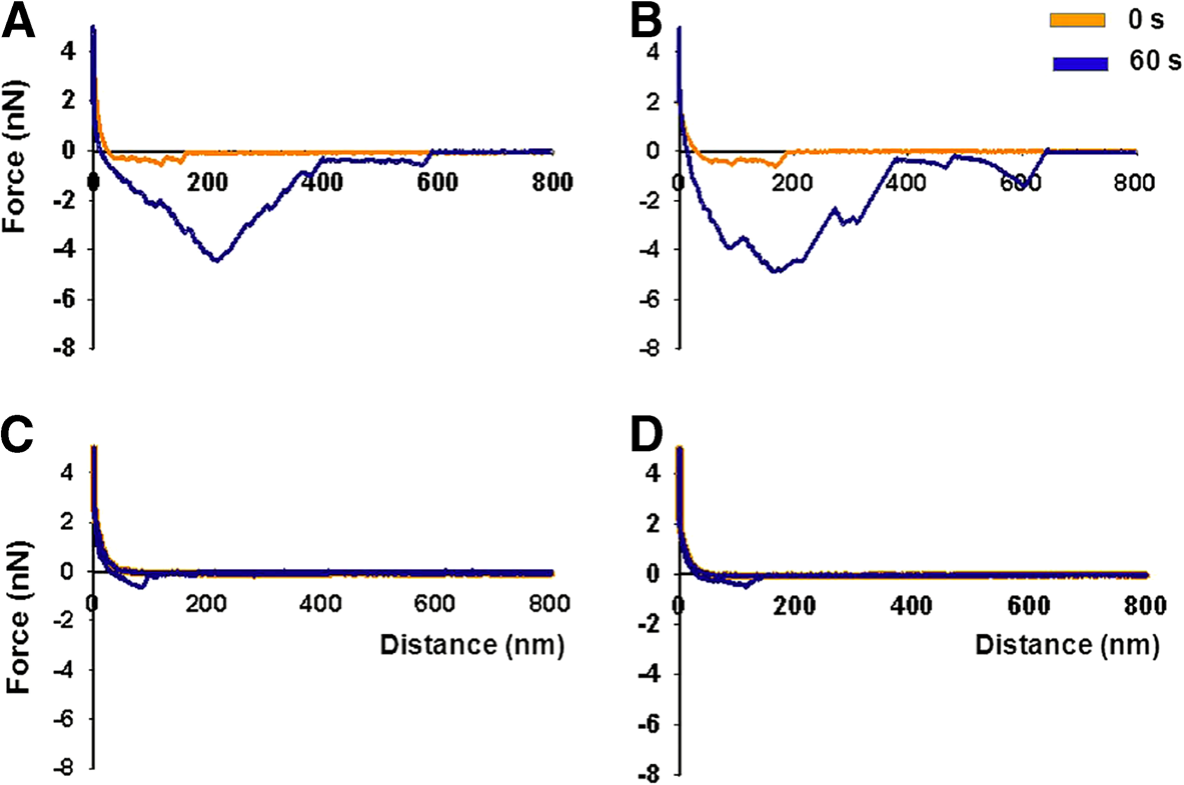
**Representative examples of force-distance curves.** Force-distance curves between different *S. aureus* NCTC8325-4^GFP^-fungus pairs upon initial contact and after 60 s bond-maturation. (**A**) *C. albicans* SC5314 hyphal tip region; (**B**) *C. albicans* SC5314 hyphal middle region; (**C**) *C. albicans* SC5314 hyphal head region; (**D**) *C. albicans* SC5314 yeast cell.

**Figure 4:**
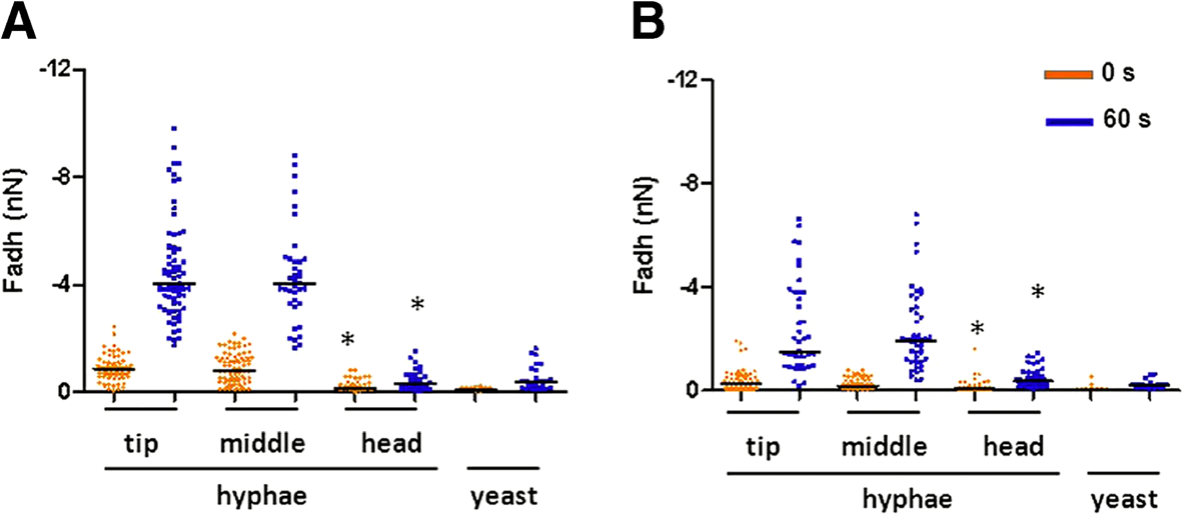
**AFM analysis of adhesion forces between *C. albicans *SC5314 and *S. aureus *NCTC8325-4**^**GFP**^**.** Vertical scatter bars of adhesion forces between *S. aureus* NCTC8325-4^GFP^ and different *C. albicans* strains and morphologies. (**A**) Different hyphal regions and yeast cells of *C. albicans* SC5314. (**B**) Different hyphal regions and yeast cells of *C. albicans* MB1. Each data point corresponds to a single force-distance curve recorded between a bacterium and a hypha. Median force values are indicated with a line. Statistically significant differences in adhesion forces (p < 0.05; Mann–Whitney test) of bacteria with the hyphal head region *versus* the middle or tip region are indicated by an asterisk.

Adhesion forces between *S. aureus* NCTC8325-4^GFP^ and both *C. albicans* strains along the hyphae were determined using AFM (Figure 1). Figure 3 shows typical examples of force-distance curves of the *S. aureus* probe upon approach and retract from *C. albicans* hyphae and yeast surfaces at initial contact and after 60 s surface delay. Major differences existed in AFM force-distance curves recorded immediately upon contact (0 s) and after a 60 s surface delay between *S. aureus* NCTC8325-4^GFP^ and different hyphal regions and the yeast cell, as summarized in Figure 4. In line with the higher number of bacteria adhering to the tip and middle regions of *C. albicans* hyphae (Figure 2C), stronger adhesion forces (around 4 nN for SC5314 and around 2 nN for MB1) were recorded after bond-maturation between these regions than for the head regions (around 0.5 nN). However, adhesion forces measured between *S. aureus* NCTC8325-4^GFP^ and both yeast cells remained comparable to the adhesion forces measured to the head region of the hyphae, irrespective of bond-maturation (Figures 4A and 4B). Note that in general, adhesion forces, especially after bond-maturation, were significantly smaller between *S. aureus* and the hyphal regions of *C. albicans* SC5314 than between *S. aureus* and *C. albicans* MB1 hyphal middle and tip regions (compare Figures 4A and 4B). 

## Discussion

In this study, we hypothesized that *S. aureus* adhesion may vary along the length of *C. albicans* hyphae. To this end, our study was designed to determine the actual physical interaction between *S. aureus* and hyphae, contingently divided into three regions, i.e. a head, middle and tip region. *S. aureus* adhered in highest numbers to the middle and tip regions of the hyphae and adhered hardly to the head region and yeast cells. In order to give new insights into this intriguing interaction, we measured staphylococcal adhesion forces directly and found that adhesion forces experienced by *S. aureus* varied along the length of *C. albicans* hyphae and were lowest in the head region of hyphae. Importantly, staphylococcal adhesion to the hyphal head region compared well with adhesion to budding yeast cells, which means that the properties of the cell wall, with respect to bacterial adhesion, remain the same for the yeast cell and head region of hyphae upon morphological change. Interestingly, electron microscopy showed that during germination, the yeast cell wall changes its morphology at the site of hyphae initiation and further formation of the germ tube requires extensive cell wall modification [30,31]. The germ-tube cell wall was not only almost two times thinner than the cell wall of the parental yeast [30,31], but also much more hydrophobic (water contact angle 107 degrees) than yeast cells (water contact angle 25 degrees) [32]. Hydrophilicity of the yeast cells is caused by the presence of mannoproteinaceous, hydrophilic, surface fibrils. Such fibrils are long, evenly spaced, radiating and mask hydrophobic proteins [33].

The biochemical composition of the cell wall of hyphae and yeast cells of *C. albicans* has been investigated extensively [34,35]. The *C. albicans* cell wall consists of two main layers: an outer layer of mannoproteins and an inner one that is composed of skeletal polysaccharides, such as chitin and β-1,3-glucans which confer strength and shape [34-36]. Although the basic cell wall components of *C. albicans* remain the same for hyphal and yeast cells, the amount and exposure of polysaccharides, as well as its surface proteome differ significantly [35-37]. For example, the amount of chitin in the hyphal cell wall is 3–5 times more than in the yeast cell wall, which could be relevant for the interaction with the host’s immune system [38]. Expression of a number of hypha-specific cell wall proteins, like agglutinin-like sequence 3 (Als3) protein, is up-regulated during the yeast-hyphae switch [37,39,40]. Als3 is specifically recognized by *Streptococcus gordonii* and allowed bacteria to adhere to the hyphae [41] and is also involved in adhesion of *S. aureus* to *C. albicans* hyphae [25]. Interestingly, Als3 protein was localized exclusively along complete hyphae and was not observed in the head region of hyphae nor in yeast cell walls [42]. This is in line with the current observation that there is no significant difference in adhesion forces between *S. aureus* and the relatively young tip region compared to older regions of the hypha.

Staphylococcal adhesion forces varied within the two *C. albicans* strains involved in this study. This effect can possibly be explained by the differential expression of cell wall associated proteins, e.g. proteins belonging to the Als family. These proteins are recognized as amyloid proteins and able to rearrange to form β-sheets, depending on environmental conditions and the strain of *C. albicans* involved [39,40,43]. Agglutinin-like sequence 3 (Als3p) is known to play a major role in the adherence process between *C. albicans* hyphae and *S. aureus*[25] and we speculate that differences in the density of Als3p along on hyphae between *C. albicans* SC5314 and MB1 account for the different adhesion forces measured with *S. aureus*. This speculation is supported by the increases in adhesion forces observed after 60 s surface delay, that may correspond to unzipping and rearrangement of a β-sheet-rich amyloid fibres [44].

## Conclusion

The findings generated from this study quantified *S. aureus* - *C. albicans* interactions and demonstrated that the head region of the hyphae is different from other hyphal regions. Therewith this study combines microbiology and physical-chemistry to yield a better understanding of the fast developing field of interkingdom interactions.

## Competing interests

The authors declare that they have no competing interest.

## Authors’ contributions

ESO, BPK, HJB, HCM designed the experiment, ESO performed the experiments, ESO, BPK, HJB, HCM analyzed the data and wrote the paper. All authors read and approved the final manuscript.
